# Research progress of hydrogels as delivery systems and scaffolds in the treatment of secondary spinal cord injury

**DOI:** 10.3389/fbioe.2023.1111882

**Published:** 2023-01-18

**Authors:** Haichuan Peng, Yongkang Liu, Fengfeng Xiao, Limei Zhang, Wenting Li, Binghan Wang, Zhijian Weng, Yu Liu, Gang Chen

**Affiliations:** ^1^ Guangdong Provincial Key Laboratory of Tumor Interventional Diagnosis and Treatment, Zhuhai People’s Hospital (Zhuhai Hospital Affiliated with Jinan University), Zhuhai, China; ^2^ The Department of Cerebrovascular Disease, Zhuhai People’s Hospital (Zhuhai Hospital Affiliated with Jinan University), Zhuhai, China; ^3^ Zhuhai Precision Medical Center, Zhuhai People’s Hospital (Zhuhai Hospital Affiliated with Jinan University), Zhuhai, China; ^4^ The Department of Neurosurgery, Zhuhai People’s Hospital (Zhuhai Hospital Affiliated with Jinan University), Zhuhai, China

**Keywords:** secondary spinal cord injury, microenvironment, delivery system, hydrogel, scaffold

## Abstract

Secondary spinal cord injury (SSCI) is the second stage of spinal cord injury (SCI) and involves vasculature derangement, immune response, inflammatory response, and glial scar formation. Bioactive additives, such as drugs and cells, have been widely used to inhibit the progression of secondary spinal cord injury. However, the delivery and long-term retention of these additives remain a problem to be solved. In recent years, hydrogels have attracted much attention as a popular delivery system for loading cells and drugs for secondary spinal cord injury therapy. After implantation into the site of spinal cord injury, hydrogels can deliver bioactive additives *in situ* and induce the unidirectional growth of nerve cells as scaffolds. In addition, physical and chemical methods can endow hydrogels with new functions. In this review, we summarize the current state of various hydrogel delivery systems for secondary spinal cord injury treatment. Moreover, functional modifications of these hydrogels for better therapeutic effects are also discussed to provide a comprehensive insight into the application of hydrogels in the treatment of secondary spinal cord injury.

## 1 Introduction

Secondary spinal cord injury (SSCI) occurs in a short time after spinal cord injury (SCI), accompanied by a series of pathophysiological reactions due to the displacement of vertebral fractures and persistent hematoma compression (Deng et al., 2018). Vascular system disorders are obvious characteristics of SSCI and ischemia-reperfusion injury (IRI) is one of those disorders, which can lead to endothelial dysfunction and vascular permeability changes. In fact, with endothelial cell injury and steady-state failure, IRI triggers a comprehensive inflammatory cascade reaction caused by the activation of residential innate immune cells (microglia and astrocytes) and infiltrating leukocytes (neutrophils and macrophages). These inflammatory cells release neurotoxins [proinflammatory cytokines and chemokines, free radicals, excitatory toxic amino acids, and nitric oxide (NO)], all of which form an inhibitory cellular growth microenvironment (Anwar et al., 2016). Hence, SSCI persists for a long time and seriously affects the quality of life of patients owing to motor and sensory dysfunction. Clinically, drugs are often used to treat SSCI after decompression of the spinal cord by surgery. Commonly used drugs include analgesics (Levendoglu et al., 2004), anti-inflammatory drugs (Hayta and Elden, 2018), and neurotrophic drugs (Nagoshi et al., 2015). The main function of drugs is to delay the progression of SSCI, inhibit the inflammatory microenvironment, and promote the differentiation and regeneration of nerve cells. However, the biggest limitation of drug treatment is that the drug cannot be directly delivered to the site of SCI. For example, after the drug is injected into a blood vessel, only a few parts can reach the site of SCI, which greatly reduces the therapeutic effect of the drug. Stem cell therapy is another important treatment option for SSCI (Mothe and Tator, 2012). Stem cells are delivered to the SCI site and promoted to differentiate into nerve cells, which is expected to restore some functions of the spinal cord. Similarly, stem cells require precise local delivery and a matrix to maintain normal cell growth and differentiation. Consequently, in the treatment of SSCI, an excellent delivery system can greatly enhance the therapeutic effects of stem cells, drugs, and other bioactive substances.

**FIGURE 1:**
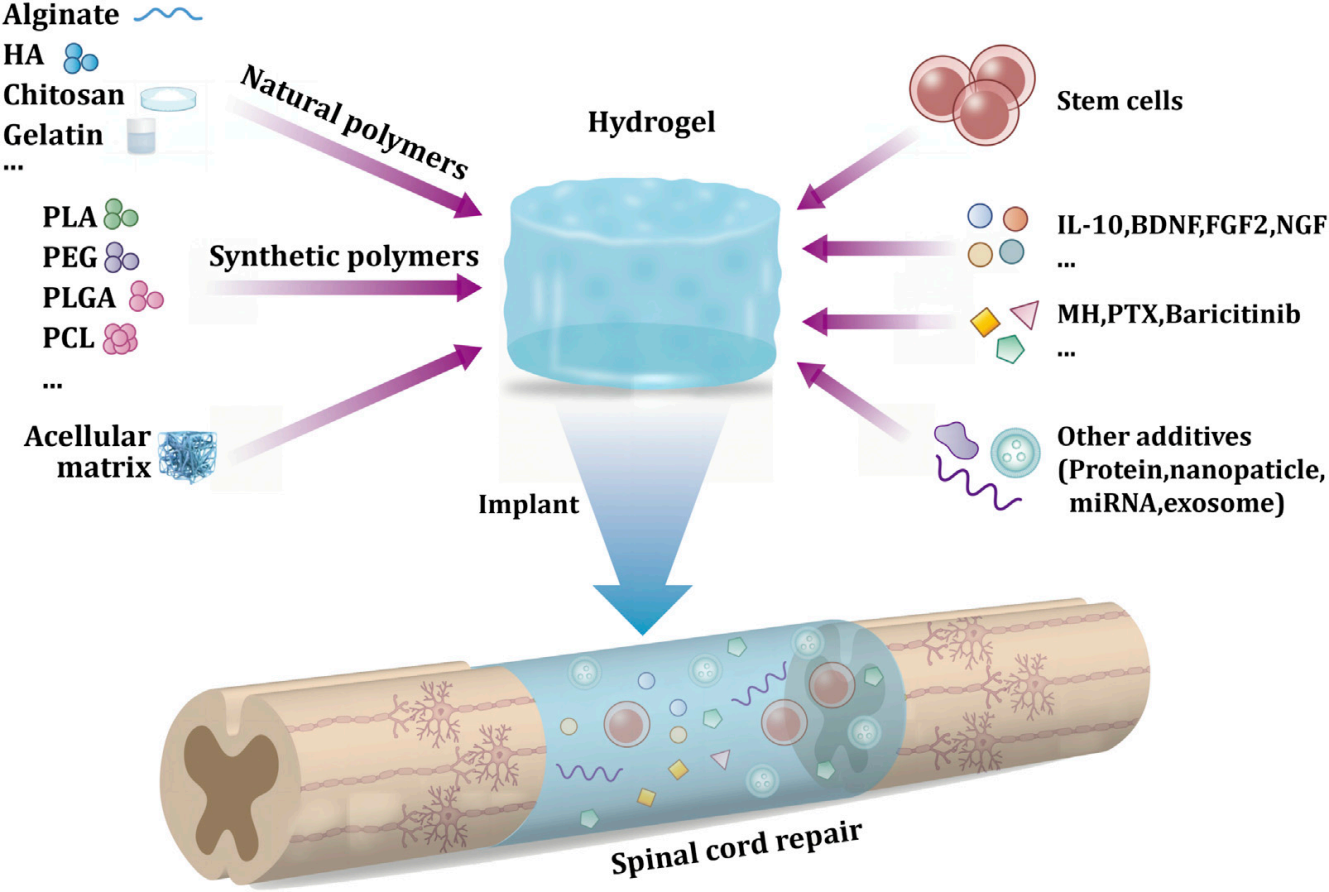
Schematic diagram of hydrogel delivery system in secondary spinal cord injury implantation treatment.

Hydrogels are biological materials similar to “jelly,” which can easily load drugs, cells, or other bioactive substances (Mantha et al., 2019; Khayambashi et al., 2021). In the hydrogel fabrication process, bioactive substances can be mixed with raw materials. Hydrogels can retain the activity of these bioactive substances. When hydrogels are transplanted into the site of SCI, these bioactive materials perform their corresponding biological functions. In addition to the load-bearing capacity of the hydrogel, it also offers several other advantages in SSCI treatment. First, a hydrogel can be prepared with excellent biocompatibility based on its raw material (Hejcl et al., 2008). Second, the mechanical properties of the hydrogel are similar to those of soft tissue, which can be embedded in spinal cord defects (Dumont et al., 2019). Third, hydrogels can serve as scaffolds to guide cell and axon growth (Madigan et al., 2014). Most mammalian cells grow in the body by adhering to a certain substrate rather than being isolated (Bacakova et al., 2011). When the hydrogel is implanted in the body, the cells adhere to the hydrogel and grow along its direction (Figure 1).

In this review, we first introduce the pathophysiology of SSCI and conclude with three stages: Acute, subacute, and chronic. Second, hydrogels as scaffolds and delivery systems for drugs and cells in SSCI treatment have also been discussed. Hydrogels can be divided into natural and synthetic hydrogels based on the different raw materials used in the preparation. The two hydrogels have different characteristics and functions. More than that, hydrogels can be modified through physical and chemical methods which acquire some new functions. Functions such as adhesion, directional arrangement structure, electrical conductivity, and injectability can significantly enhance the ability of hydrogels as scaffolds and delivery systems. Finally, future trends of hydrogels in SSCI therapy are discussed. We hope that this review will be beneficial for the development of hydrogels for SSCI.

## 2 Pathophysiology of SSCI

**FIGURE 2:**
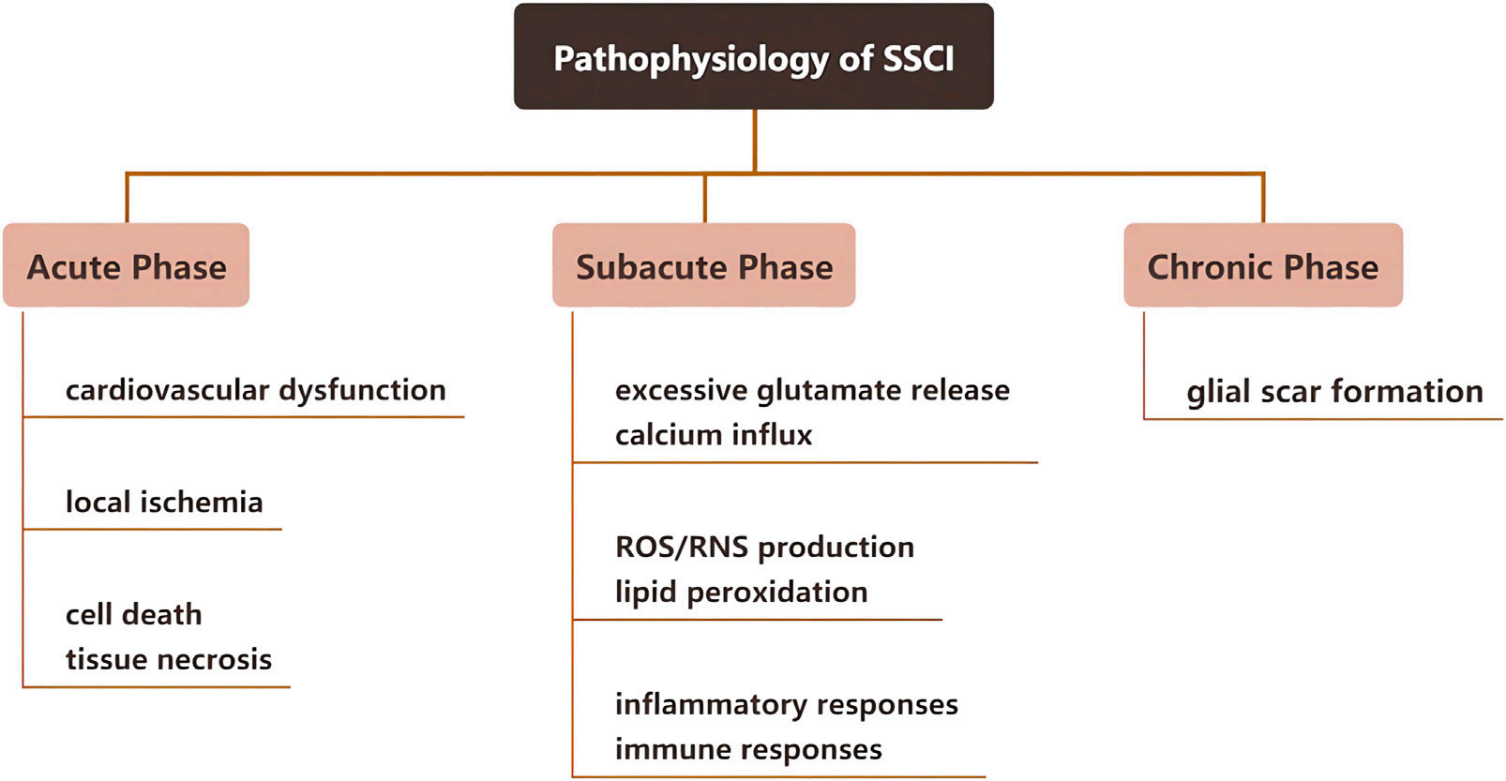
Pathophysiology, clinical manifestations, and phases of secondary spinal cord injury.

SSCI occurs within a relatively short period after the first stage of injury. Based on the pathophysiological response of SSCI, it can be divided into three stages: Acute, subacute, and chronic; the detailed physiological responses at each stage are shown in Figure 2. The first stage of injury leads to SSCI and causes chemical and mechanical injury to the spinal cord tissue. Owing to high intracellular calcium accumulation, neuronal excitotoxicity is induced, and reactive oxygen species (ROS) concentration and glutamate levels are increased. The clinical manifestations of SSCI include vascular damage and ischemia, edema, excitotoxicity, ionic dysregulation, inflammation, lipid peroxidation, free radical production, demyelination, glial scarring, and cyst formation (Walsh et al., 2022). Research has indicated that SSCI forms an inhibitory microenvironment that hinders the regeneration of nerve cells (Couillard-Despres et al., 2017; Anjum et al., 2020).

### 2.1 Acute phase of SSCI

Depending on the degree of injury in the acute phase of SSCI, nerve-dominated respiratory and cardiovascular functions may be affected, thereby affecting gas exchange and oxygen transport in the spinal cord (Berlly and Shem, 2007). Moreover, destruction of the microvascular system will first lead to bleeding and edema in a few seconds and then damage the blood perfusion of the injured spinal cord, leading to thrombosis and vasospasm. It further aggravates local ischemia and ultimately leads to cell death and tissue necrosis (Witiw and Fehlings, 2015). Furthermore, in pathological conditions such as nervous system ischemia, trauma, and degenerative changes, glutamate can mediate neurotoxicity through excitatory glutamate receptors, resulting in neurological dysfunction (Nishizawa, 2001).

### 2.2 Subacute phase of SSCI

After acute SSCI, spinal cord ischemia and hematoma lead to a series of changes in the tissues and cells. For example, spinal cord ischemia can lead to excessive glutamate release, resulting in cellular and tissue toxicity (Hermann et al., 2001). As byproducts of cell necrosis (DNA, ATP, K^+^) are released into the microenvironment after injury, activated microglia rapidly secrete inflammatory cytokines, leading to peripheral immune cell infiltration through the damaged blood-spinal cord barrier. This leads to a periodic increase in chemical attractants and activated immune cells, which can spread local inflammation within weeks to months of injury. The inflammatory response of SSCI involves a variety of cellular components, such as microglia, neutrophils, and macrophages, and molecular components, such as cytokines, prostaglandins, and complement systems.

### 2.3 Chronic phase of SSCI

In the chronic stage of SSCI, a large number of proliferating astrocytes migrated to the SCI area and interlaced with fibroblasts invading the lesion area to form glial scars. Glial scar formation is usually referred to as reactive astrocyte proliferation, including astrocyte hypertrophy, proliferation, migration, and upregulation of glial fibrillary acidic protein (GFAP), vimentin, and nestin expressed by astrocytes (Karimi-Abdolrezaee and Billakanti, 2012). Astrocytes play a crucial role in maintaining homeostasis of the central nervous system (CNS), regulating ion concentrations, providing metabolic support for adjacent neurons, stabilizing synapses, and supporting the neurovascular system, including maintaining the blood-brain barrier (BBB) and producing the extracellular matrix (ECM). After CNS injury, astrocytes are activated to reactive astrocytes. Reactive astrocytes at the lesion site and surrounding areas immediately proliferate resulting in morphological changes, which upregulate the production of extracellular proteins and eventually form glial scars. Glial scars inhibit nerve regeneration, but the underlying mechanism is not clear. Some researchers have found that tumor necrosis factor-inducible gene 6 protein (TSG-6) secreted by astrocytes is involved in glial scar formation and inhibits inflammation (Coulson-Thomas et al., 2016; Day and Milner, 2019). Additionally, the main reason for the non-regeneration of damaged axons is the deposition of chondroitin sulfate and keratin sulfate proteoglycan secreted by reactive astrocytes in glial scars (Vadivelu et al., 2015). Although most studies have demonstrated that glial scars have adverse effects on nerve regeneration, some studies have shown that glial scars play an important role in rapidly repairing the blood-brain barrier and reducing inflammatory cell infiltration and neuronal degeneration, thereby reducing the harmful effects of injury (Faulkner et al., 2004). It is worth mentioning that Anderson et al. demonstrated in gene knockout mice that inhibiting the formation of glial scar did not stimulate axonal regeneration, but to a certain extent, the glial scar could help axonal regeneration, which may be related to the expression of multiple axon growth support molecules by astrocytes and non-astrocytes (Anderson et al., 2016).

## 3 Treatment strategies of hydrogels as scaffolds and delivery systems for SSCI

**FIGURE 3:**
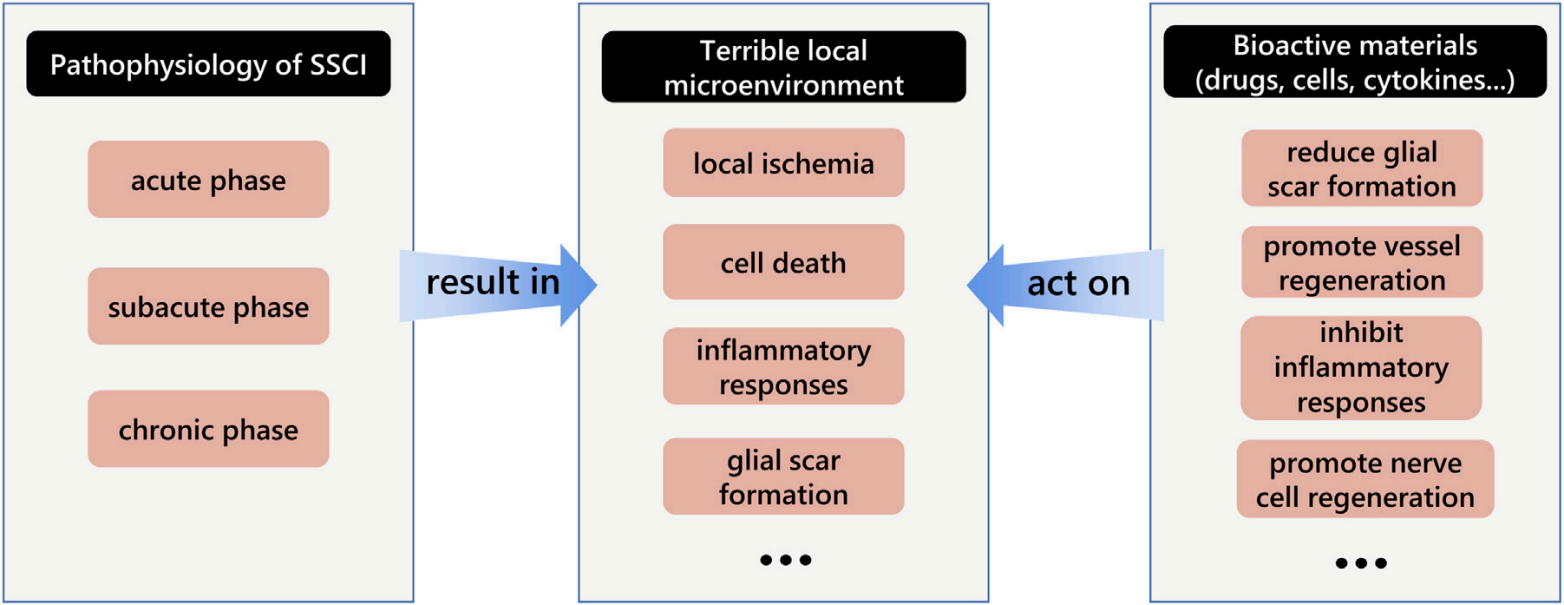
Schematic diagram of the interaction among the pathophysiology of secondary spinal cord injury, terrible local microenvironment and bioactive materials.

The pathophysiology of SSCI influences the treatment strategies. SSCI is accompanied by a series of microenvironment changes such as microvascular system destruction, nerve cell death, and inflammatory responses (Anwar et al., 2016). In current clinical treatment strategies, aggressive intensive care measures are essential in the acute stage, including early surgical decompression, treatment with anti-inflammatory drugs, blood pressure augmentation, and stabilization of respiratory and cardiac complications (Karsy and Hawryluk, 2019). In subacute and chronic stages, rehabilitation and medication are the main treatments (Huang et al., 2019). Commonly used drugs include vitamins (Hosseini et al., 2020), gangliosides, and other neurotrophic factors (Kim et al., 2017; Pinelli et al., 2022). In addition, some of the latest treatments, such as cell transplantation and cells with scaffold transplantation therapies, are also performed in the chronic stage (Fan et al., 2017; Chen et al., 2021). The main goal of treatment is to improve the terrible microenvironment at the site of SCI and promote neuronal growth and differentiation, which has also been demonstrated in recent studies (Ashammakhi et al., 2019; Bellák et al., 2020) (Figure 3). For example, cytokines such as fibroblast growth factor-2 (FGF2) (Xu et al., 2018), glial cell-derived neurotrophic factor (GDNF) (Zhu et al., 2015), basic fibroblast growth factor (bFGF) (Wang et al., 2021a), brain-derived neurotrophic factor (BDNF), and vascular endothelial growth factor (VEGF) (Wen et al., 2016) have often been used for SSCI treatment. These active materials are beneficial to nerve cell growth and vessel regeneration. However, delivery into the spinal cord remains a problem (Rossi et al., 2013). Hydrogels are widely used in the medical field because of their excellent biocompatibility, mechanical properties similar to soft tissue, and strong load-bearing capacity, and have been used to load a variety of drugs, cells, and cytokines to the spinal cord to achieve therapeutic effects (Silva et al., 2021). Moreover, hydrogels can be used as cell scaffolds to guide the axon regeneration of nerve cells. Recently, hydrogels have been used as delivery systems for SSCI. Hydrogels are divided into two groups according to the raw materials used: natural and synthetic hydrogels (Xie et al., 2022). The former is composed of natural polymers, such as polysaccharides and peptides, while the latter is composed of synthetic hydrophilic polymers, including alcohols, acrylic acids, and their derivatives. Moreover, hydrogels can be modified to acquire specific functions such as adhesion and conductivity, which are beneficial to their delivery ability. In addition, with the advancement of this technique, new methods such as 3D printing or electrospinning for hydrogel fabrication have been applied to design hydrogels. Their advantages include faster synthesis efficiency, more regular and uniform internal structures, and suitability for mass synthesis, providing another path for future application in the treatment of SSCI.

### 3.1 Natural hydrogels for the treatment of SSCI

**FIGURE 4:**
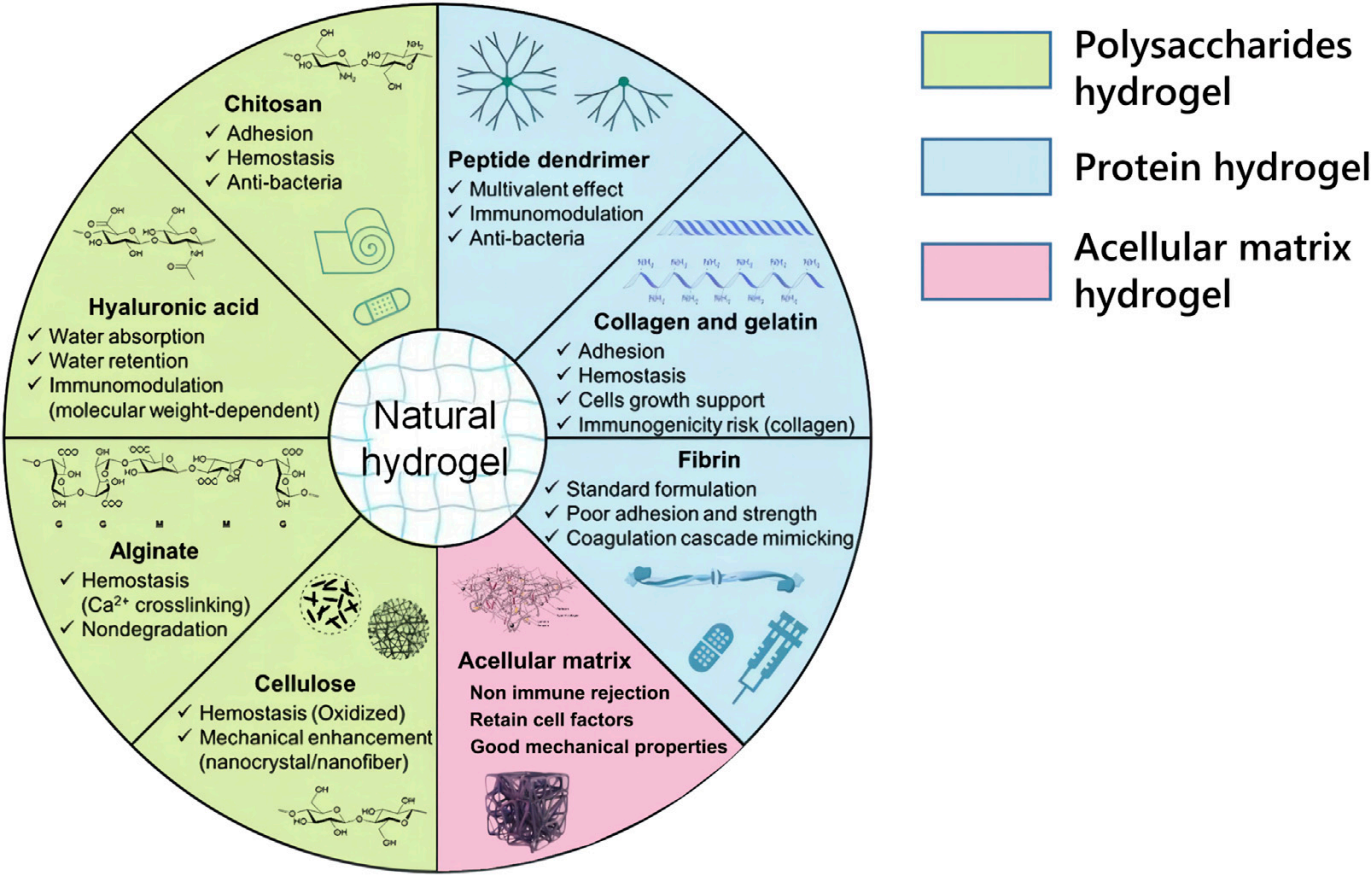
Schematic diagram of commonly used natural hydrogels and their biological functions.

Natural hydrogels mainly include three categories: proteins (collagen, gelatin, fibrin, etc.), polysaccharides (hyaluronic acid, chitosan, heparin, starch, cellulose, alginate, etc.), and acellular matrices (Catoira et al., 2019; Wu et al., 2022) (Figure 4). The three types of natural hydrogels have inherent biocompatibility and bioactivity, which are suitable for biomedical applications because of their ability to promote cell functions and the fact that their structures and properties are similar to those of natural soft tissues (Zhu and Marchant, 2011).

#### 3.1.1 Protein-based hydrogel

Proteins are polymers with clearly defined sequences, and their chemical properties at the molecular level (crosslinking density and number of bioactive ligands) can be well controlled (Krishna and Kiick, 2010). Protein-based hydrogels can be composed entirely of proteins or mixed networks of proteins and other polymers. Protein-based hydrogel components are attractive because of their structural designability, specific biological functions, and stimuli responsiveness (Schloss et al., 2016). Compared with other polymers, the multifunctionality of proteins endows protein hydrogels with a variety of biofunctions. Collagen hydrogels are natural materials with excellent biocompatibility, adhesiveness, and biodegradability (Gao et al., 2018). Yang et al. (2021) designed a collagen hydrogel loaded with the small-molecule drugs LDN193189, SB431542, CHIR99021, and P7C3-A20. *In vitro* and *in vivo* experiments verified that this combined collagen hydrogel delivery system effectively promoted the differentiation of endogenous neural stem cells (NSCs) into neurons and inhibited their differentiation into astrocytes. Zhou et al. (2020) used gelatin methacryloyl (GelMA) hydrogel as a carrier to load bone mesenchymal stem cells (BMSCs) and neural stem cells (NSCs) and implanted them into animals. GelMA hydrogel is commonly used for drug delivery *in vivo* and is a photosensitive hydrogel composed of a processed natural ECM. The mechanical properties of GelMA can be changed by adjusting the hydrogel concentration, crosslinking degree, and gel time to simulate the softness of the spinal cord (Nichol et al., 2010). Owing to the structural properties of three-dimensional GelMA hydrogel, it has good permeability to oxygen and nutrients (Chen et al., 2019). GelMA hydrogels can also promote cell survival, proliferation, and migration, and NSCs differentiate into neurons, reducing the formation of astrocytes (Fan et al., 2018). Fan et al. (2022) directly loaded bone marrow stem cell-derived exosomes (BMSC-exosomes) into GelMA hydrogel scaffolds, and as the scaffolds were implanted into animals, they could enhance local NSC recruitment and promote neuronal and axonal regeneration, resulting in significant functional recovery in the early period in an SCI mouse model. Fibrin is a common raw material for protein-based hydrogels. Sudhadevi et al. (2021) synthesized a fibrin hydrogel loaded with neural progenitor cells (NPCs). Fibrin hydrogel is composed of a fibrinogen solution mixed with thrombin. The porosity, pore size, and fiber chain thickness were tuned by varying the amount of thrombin. Different thrombin contents also affect the morphology of NPCs and cell survival *in vitro*. In addition, fibrin hydrogel as a flexible scaffold can induce neural stem/progenitor cells (NSPCs) to differentiate into dopaminergic/noradrenergic neurons and synaptic protein networks (Bento et al., 2017). King et al. (2010) examined four biomaterials that can be injected into an injury site and gel *in situ*: collagen, viscous fibronectin, fibrin, and fibrin + fibronectin (FB/FN). The *in vivo* experiments were divided into four groups and injected into the knife-cut cavity in the rat spinal cord. At 1 week, all four materials showed good integration with the host spinal cord and supported some degree of axonal ingrowth. But at 4 weeks, the FB/FN mixture showed the best combination with the host spinal cord tissue and supported the robust growth of axons, which demonstrated that the mixture of fibrin and fibronectin could enhance the supporting properties of the scaffold.

#### 3.1.2 Polysaccharide hydrogel

Polysaccharides exist in organisms and can be obtained from renewable sources. Polysaccharide-based hydrogels can retain large amounts of water, are usually non-toxic and biocompatible, and possess special physical and chemical properties (Coviello et al., 2007). Kushchayev et al. (2016) demonstrated that hyaluronic acid (HA) hydrogel had a neuroprotective effect on the spinal cord by decreasing the magnitude of SSCI. It can reduce disorganized scar tissue and retain neurons near and above the lesion. Wen et al. (2016) fabricated a novel scaffold composed of an anti-Nogo receptor antibody (antiNgR)-modified HA hydrogel and poly(lactic-co-glycolic acid) (PLGA) microspheres containing BDNF and VEGF. They showed that the implants and host tissues could be well integrated and that inflammation and glial hyperplasia were inhibited. In particular, a large number of new blood vessels and regenerated nerve fibers were found inside and around the implants. Chitosan is prepared by the N-deacetylation of chitin, which is a natural polysaccharide obtained from the exoskeleton of crustaceans or the cell wall of fungi. Chitosan has excellent biocompatibility, biological activity, biodegradability, and high mechanical strength and is widely used in the field of tissue engineering (Othman et al., 2021; Yu et al., 2021; Liu et al., 2022). For example, Chedly et al. (2017) prepared physical chitosan microhydrogels as scaffolds for SSCI restoration and axon regeneration. Animal experiments showed that chitosan scaffolds could promote the regeneration of axons, and growing axons were myelinated or ensheathed by endogenous Schwann cells that migrated into the lesion site. Moreover, the chitosan hydrogel could regulate the inflammatory response at the injury site and improve the local microenvironment. Mahya et al. (2021) used chitosan and alginate as raw materials to crosslink and synthesize composite hydrogels containing berberine-loaded chitosan nanoparticles. The mixing of chitosan nanoparticles and alginate can regulate the swelling and degradation of hydrogels, thereby providing a suitable microenvironment for spinal cord tissue engineering. Huang et al. (2021) designed a thermosensitive quaternary ammonium chloride chitosan/*β*-Glycerophosphate (HACC/β-GP) hydrogel scaffold combined with BMSCs. BMSCs were transfected with adenovirus containing GDNF gene. GDNF can promote the differentiation of BMSCs into neurons (Zhu et al., 2015), whereas HACC can provide a non-toxic microenvironment that supports cell adsorption and growth. Heparin is another raw material commonly used for polysaccharide hydrogels. Heparin-based hydrogels are widely used in various applications, including implantation, tissue engineering, biosensors, and drug-controlled release, owing to the 3D constructs of hydrogels (He et al., 2019a). Albashari et al. (2020) reported a injectable heparin hydrogel loaded with bFGF and dental pulp stem cells (DPSCs). This team injected it into spinal cord defects of Sprague-Dawley rats. Animal experiments have shown that the system could regulate the inflammatory response, accelerate nerve regeneration, and inhibit the proliferation and activation of microglia/macrophages. Another common raw material for polysaccharide hydrogels is alginate, which can be prepared into hydrogels for cell culture (Andersen et al., 2015; Neves et al., 2020). Liu et al. (2017) transplanted Schwann cell-seeded alginate capillary hydrogels into a spinal cord lesion site. With adeno-associated viral 5 (AAV5) expressing BDNF injected and activated caudally, the number of regenerating axons significantly increased.

#### 3.1.3 Acellular matrix hydrogel

The cellular matrix comprises all components of the tissue, except cells, including a homogeneous matrix (proteoglycans and glycoproteins) and filamentous collagen fibers. It removes antigen components that can cause immune rejection after the acellular processing of allogeneic tissues while completely retaining the three-dimensional structure of the ECM and some growth factors that play an important role in cell differentiation, such as FGF2, transforming growth factor β (TGF-β), and VEGF (Badylak et al., 2011). The treated ECM material has excellent mechanical properties and histocompatibility that can support and connect cells *in vivo*, without immune rejection (Liang et al., 2020). Simultaneously, its 3D spatial structure and cytokines are conducive to cell adhesion and growth. Xu et al. (2021) compared the biological functions of a decellularized tissue matrix hydrogel derived from the spinal cord (DSCM-gel) and a decellularized matrix hydrogel derived from the peripheral nerve (DNM-gel). The results showed that the hydrogel processed by DSCM had a nano-fiber structure simulating the ECM and a relatively larger pore size. DSCM-gel provides a regenerative 3D microenvironment that enhances the survival, proliferation, and migration of NSCs/NSPCs), and is followed by a unique ability to promote neuronal differentiation and synapse formation in NSPCs.

Natural hydrogels can be loaded with drugs, cytokines, miRNAs, stem cells, and stem cell-derived exosomes for local delivery to treat SSCI. Most functional additives are loaded physically. However, they have different requirements in different situations (Chai et al., 2017). For drug loading, no substances affecting drug activity should be added during the preparation of hydrogels, and the temperature should also be within the appropriate range (Li and Mooney, 2016). Moreover, there are some limitations in delivering incompatible hydrophobic drugs. In order to address these problems, different strategies have been employed to modify the hydrogel system, including the introduction of hydrophobic domains, the addition of nanoparticles, and inclusion of cyclodextrin (CD) groups (Gu et al., 2017). For cytokine loading, a suitable temperature is required during the loading process, which cannot be inactivated. Therefore, there are certain requirements for the hydrogel type and preparation method (Schirmer et al., 2016). Stem cells and stem cell-derived exosomes have high requirements for the microenvironment of hydrogels because the survival of stem cells and exosomes requires sufficient nutritional support (Khayambashi et al., 2021). Stem cells and exosomes can be prepared in suspension together with the raw materials of the hydrogel to form an injectable hydrogel. They can also be co-cultured with hydrogel scaffolds for a certain period. After cell growth, differentiation, and adhesion to the hydrogel scaffold, it was implanted into the body to achieve spinal cord delivery (Albashari et al., 2020; Huang et al., 2021). Stem cell-derived exosomes have characteristics of stem cells, such as directional migration homing ability and low immunogenicity. Studies have shown that stem cell-derived exosomes can effectively transport bioactive substances, such as mRNA, microRNA, and protein, and have important biological functions, such as reducing apoptosis, reducing the inflammatory response, promoting angiogenesis, inhibiting fibrosis, and improving tissue repair potential (Khalatbary, 2021). Some recent studies on natural hydrogel-loaded different types of stem cells and substances that assist nerve cell differentiation and growth are summarized in Table 1 (Li et al., 2016; Thompson et al., 2018; He et al., 2019a; Yao et al., 2021; Chen et al., 2022a). During SSCI, endogenous NSCs can grow and differentiate, however, most differentiate into astrocytes rather than neurons without intervention. Hydrogels and drugs have the potential to recruit endogenous NSCs [(Lau and Wang, 2011; Chen et al., 2022a; Zabarsky et al., 2022), thus some researchers have focused on promoting the differentiation of endogenous NSCs into neurons by loading drugs or bioactive substances on hydrogels. Some drugs and bioactive substances used by researchers in recent years and their biological functions are listed in Table 2 (Zhao et al., 2016; Wang et al., 2017a; Xu et al., 2018; He et al., 2019b; Han et al., 2020; Nazemi et al., 2020; Raspa et al., 2021; Zhang et al., 2021; Gao et al., 2022; Shen et al., 2022; Xu et al., 2022).

**TABLE 1:** Some natural hydrogels loaded with stem cells for the treatment of secondary spinal cord injury.

Hydrogel composition	Loaded stem cells	Results	Reference
·Gelatin	·Human umbilical cord mesenchymal stem cells (hUC-MSCs)	·*In vitro*, hUC-MSCs in 3D gelatin hydrogel displayed good viability, proliferation, and neuronal differentiation	Yao et al. (2021)
·Hydrogen horseradish peroxidase (HRP)		·*In vivo*, promoted motor function recovery, decreased inflammation, inhibited apoptosis and promoted neurogenesis	
·Galactose oxidase (GalOx)			
HA/ECM harvested from protoplasmic (grey matter) astrocytes	·Mouse embryonic stem cells-derived V2a interneurons	·ECM implantation was found to alter the behavior of immune cells, astrocytes, and neurons within the context of the injured spinal cord	Thompson et al. (2018)
		·The HA hydrogels were also found to support the transplantation of V2a interneurons into the SCI lesion	
·Methacrylamide chitosan	·Adult neural stem or progenitor cells	·Significant reduction in lesion area and macrophage infiltration around the lesion site	Li et al. (2016)
·Interferon-γ (IFN-γ) platelet-derived growth factor-AA (PDGF-AA)			
·Gelatin methacrylate	·Induced pluripotent stem cells (iPSCs)-derived NSCs (iNSCs)	·Decreased inflammation by reducing activated macrophages/microglia (CD68-positive cells)	He et al. (2019a)
·Irgacure 2959		·Inhibiting GFAP-positive cells and glial scar formation	
·Collagen	—	·Promoting axonal regeneration	Chen et al. (2022a)
·Fibrin		·Induced endogenous NSPC migration to the lesion site	
·Stromal cell-derived factor-1α (SDF1α)		·Promoted neuronal differentiation of the recruited NSPCs	
·Paclitaxel (PTX)			

**TABLE 2:** Some bioactive substances loaded into natural hydrogels and their biological functions.

Hydrogel	Bioactive compounds	Biological functions	Reference
·Hyaluronan-methylcellulose (HAMC)	·Brain-derived neurotrophic factor(BDNF)	·Promoting axon regeneration	He et al. (2019b)
·Heparin-poloxamer (HP)	·Fibroblast growth factor-2(FGF2)	·Neuroprotective and neurotrophic, inhibiting excessive astrogliosis and glial scarring	Wang et al. (2017a), Xu et al. (2018)
·Gelatin	·Interleukin-10 (IL-10)	·Suppressing monocyte/macrophage inflammatory response, regulating microglia and macrophage M2-polarization, and promoting neuronal cell survival	Shen et al. (2022)
·Alginate	·Minocycline hydrochloride (MH)/paclitaxel (PTX)	·Neuroregenerative and neuroprotective drug	Nazemi et al. (2020)
·Silk protein nanofiber	·Nerve growth factor (NGF)	·Regulated the neuronal/astroglial differentiation of neural stem cells	Zhao et al. (2016), Gao et al. (2022)
·HP			
·Glycol chitosan-oxidized hyaluronate	·Tauroursodeoxycholic Acid	·Anti-neuroinflammatory	Han et al. (2020)
·Aligned electrospun fibers	·Axon microRNAs/methylprednisolone	·Decreases the expression of pro-inflammatory genes, promotes functional recovery and remyelination	Zhang et al. (2021)
·HAMC	·Fat extract	·Promotes the polarization of macrophages from an inflammatory M1 phenotype to an anti-inflammatory M2 phenotype	Xu et al. (2022)
·Self-assembling peptide	·Chondroitinase ABC (ChABC)	·A thermolabile pro-plastic agent attenuating the inhibitory action of chondroitin sulfate proteoglycans	Raspa et al. (2021)

### 3.2 Synthetic hydrogels for the treatment of SSCI

Synthetic hydrogels are easier to modify than natural hydrogels. Some synthetic hydrogels exhibit slow degradation, thus providing excellent durability for biomaterials (Garnica-Palafox and Sanchez-Arevalo, 2016). Synthetic hydrogels are fabricated by physical or chemical cross-linking of synthetic polymers, including polyvinyl alcohol (PVA), poly(ε-caprolactone) (PCL), PLGA, polyoxyethylene, polyethylene glycol (PEG), acrylic acids, and their derivatives (Gyles et al., 2017). Based on the types of raw materials used, we classified and described the synthetic hydrogels as follows.

#### 3.2.1 PVA

Chen et al. (2022b) designed a hydrogel based on PVA and mixed it with molybdenum disulfide/graphene oxide (MoS2/GO). Studies have shown that compound hydrogels containing 10% (w/v) PVA exhibit the closest strength to the spinal cord and good adhesion. Hiraizumi et al. (1995) used PVA hydrogel membranes after spinal surgery at L5 in 30 adult cats. *In vivo* experiments demonstrated that PVA hydrogel membranes prevented the migration of inflammatory cells, thereby reducing the formation of intraspinal scar tissue and adhesion reactions. Other beneficial features of PVA include extreme resilience and low friction, eliminating the mechanical response to the spinal cord.

#### 3.2.2 PCL

Nisbet et al. (2008) developed a PCL scaffold by electrospinning. PCL scaffolds were modified with ethylenediamine (ED), which demonstrated that electrospun PCL can direct the differentiation of NSCs towards a specific lineage. Similarly, Yen et al. (2019) prepared novel electrospun PCL/type I collagen nanofiber conduits for SSCI repair. *In vivo* experiments showed that the nanofiber conduit had the potential to repair sciatic nerve defects. It was also observed that PCL/type I collagen nanofiber conduits with excellent biocompatibility.

#### 3.2.3 PLGA

Another synthetic polymer, PLGA, was used by Zheng et al. (2021) to fabricate thermoreversible hydrogels. When PLGA is copolymerized with PEG, it can acquire a solid-liquid transition at physiological temperature (Wang et al., 2017b). PLGA/PEG block thermoreversible gelling polymers are widely studied hydrogels for drug delivery owing to their biodegradability and excellent biocompatibility (Chen et al., 2017). Meanwhile, baricitinib could be mixed into the PLGA-PEG-PLGA solution and injected into the site of SCI. The results showed that the hydrogel solution could gel in the body, disintegrate within 72 h, and release the drug.

#### 3.2.4 PNIPAAm

Similar to the aforementioned PLGA-PEG-PLGA hydrogel, Bonnet et al. (2020) designed a poly (N-isopropyl acrylamide) (PNIPAAm)-polyethylene glycol (PEG) hydrogel. It has been demonstrated that PNIPAAm can form a hydrogel with thermosensitive and solid-liquid conversion properties (Klouda et al., 2011), and its universal conversion temperature is 32°C; however, when PNIPAAm is copolymerized with a hydrophilic polymer such as PEG, its solid-liquid conversion temperature can be increased to 37°C. In addition, their mechanical and viscoelastic properties can be tailored (Vernengo et al., 2008). Experiments showed that PNIPAAm-g-PEG improved sensory and motor recovery, and supported axonal regeneration.

#### 3.2.5 PEG

It is usually used as a matrix for preparing hydrogels because it has the following advantages. First, in the acute phase of SCI, it can resist nerve fiber degeneration, reduce inflammation, inhibit vacuole, and scar formation, and protect the nerve membrane. Second, PEG coupling polymers can not only promote angiogenesis but also transport drugs or bioactive molecules to the injured site. Third, PEG hydrogels can be used as supporting substrates for stem cell growth after injury to induce cell migration, proliferation, and differentiation (Kong et al., 2017). Typically, PEG must be combined with other polymers to form a hydrogel system. Zhang et al. (2020a) designed a conductive hydrogel by combining graphene oxide and PEG. Hydrogels have good recoverability and injectability, allowing drugs to be injected *in situ* into the site of SCI and realizing injury repair.

#### 3.2.6 Acrylic acids and their derivatives

Acrylic acids and their derivatives, such as polyacrylamide, can be used to fabricate hydrogel delivery systems. Dong et al. (2020) used polyacrylamide as a raw material to synthesize a conducting polymer hydrogel (CPH). *In vitro* experiments showed that near-infrared light irradiation enhanced the conductivity of the CPH, thereby promoting the conduction of bioelectrical signals. When the CPH is mechanically elongated, it still has high conductive durability, which can accommodate the unexpected strain of nerve tissue in motion. Wang et al. (2021b) prepared an F127-polycitrate-polyethyleneimine hydrogel (FE) loaded with extracellular vesicles for SSCI treatment. This delivery system was shown to inhibit fibrosis scar formation, reduce inflammation, promote myelin and axon regeneration, and synergistically induce effective spinal cord treatment. F127 is a copolymer of polycitrate-polyethyleneimine, which is rapidly crosslinked and solidified into a gel by ultraviolet (UV) and visible light in the presence of a photoinitiator. F127 has excellent thermo-induced gel characteristics and good biosafety, and polycitrate-polyethyleneimine can be loaded with extracellular vesicles through electrostatic interactions. The team prepared thermosensitive and injectable hydrogels based on the characteristics of these two materials, which greatly improved the possibility of their practical application in the treatment of SSCI.

### 3.3 Functionalized hydrogels for SSCI treatment

In recent research, there is a lot of evidence that revealed that hydrogels with special functions could strengthen the delivery ability and further enhance the therapeutic effect (Walsh et al., 2022). The directional arrangement structure, electrical conductivity, high adhesion, antioxidant capacity (Zhang et al., 2020b), mechanical strength (Zhai et al., 2020), and injectability (Wang et al., 2018) of hydrogels are of great significance for improving the microenvironment in the pathophysiology of SSCI. Several commonly used functions for hydrogel modification are described below:

#### 3.3.1 Directional arrangement structure

**FIGURE 5:**
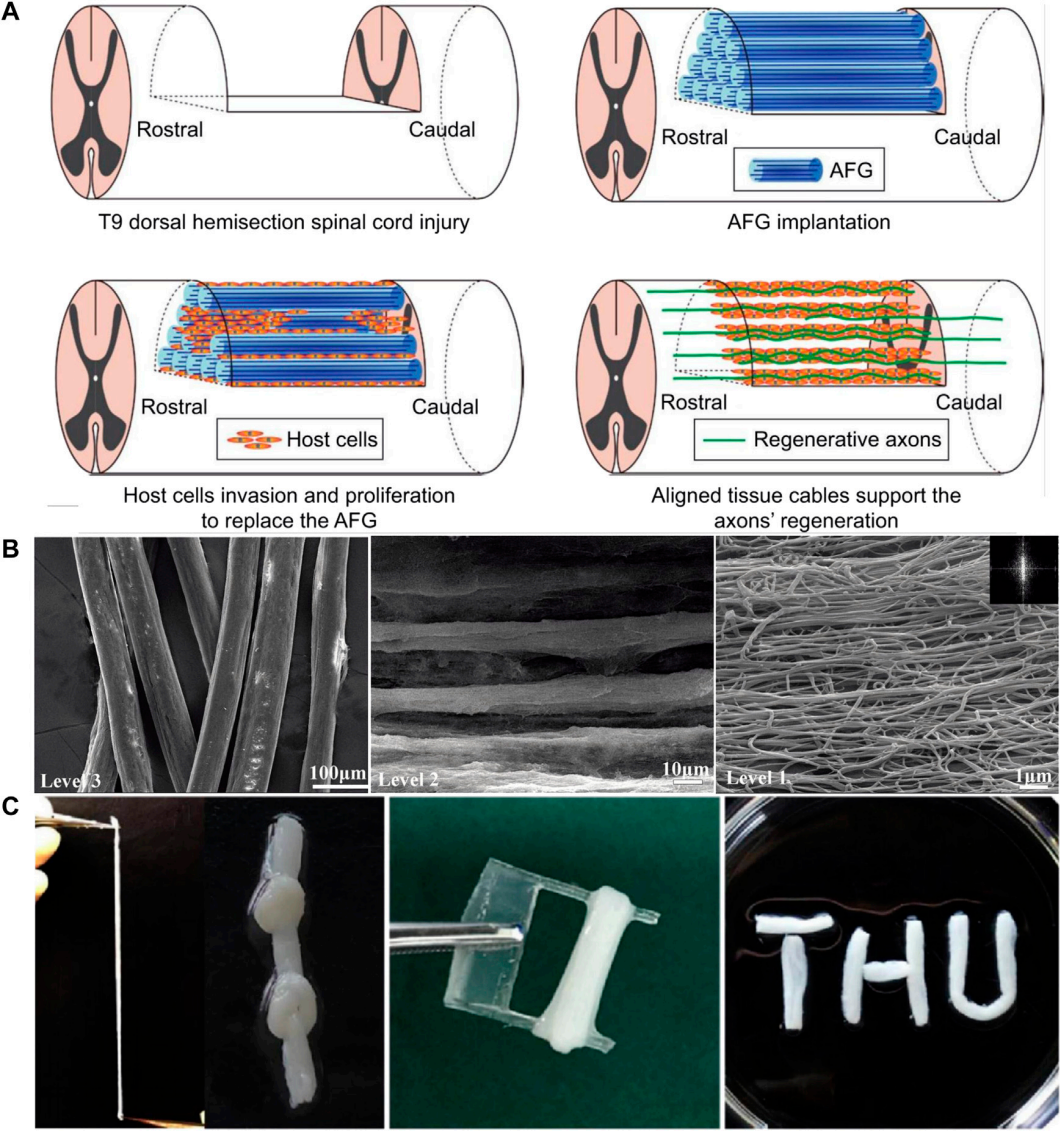
Characteristics of aligned fibrin hydrogel. **(A)** Schematic illustration of the mechanism for the axonal regrowth process with the involvement of an aligned scaffold. Taken from (Yao et al., 2018). **(B)** SEM images of AFG at different magnifications showing hierarchically aligned organizations. [Reprinted with permission from Ref. (Yao et al., 2016). Copyright 2016 Royal Society of Chemistry]. **(C)** Mechanical characters of the AFG. The AFG can be stretched, knotted, twined, and clipped. [Reprinted with permission from Ref. (Cao et al., 2020). Copyright 2020 Springer Nature].

Hydrogels with regularly oriented structures can act as scaffolds to support cell growth and guide axon regeneration. Yao et al. (2020) prepared an aligned fibrin hydrogel (AFG) as a cell growth scaffold to guide nerve cell growth. Stem cells were transplanted into the AFG, and the scaffolds were implanted into animals. AFG scaffolds can induce host nerve cells to migrate to hydrogels, which can replace the scaffold structure to form aligned cell fibers and create a bioactive environment for nerve fiber regeneration *in vivo*. It was worth mentioning that AFG has low elasticity which can better fit the soft tissue of the spinal cord. More importantly, the structure of the AFG directional arrangement was conducive to the directional growth of axons (Zhang et al., 2017; Yao et al., 2018) (Figure 5A). Yao et al. (2016) designed an AFG loaded with human umbilical mesenchymal stem cells (hUMSCs), and scanning electron microscopy (SEM) images showed that the AFG had regularly aligned internal structures at different magnifications (Figure 5B). *In vitro* experiments verified that hUMSCs can grow and differentiate into oriented fibrin hydrogels and form new directional axons. Four weeks after implantation of this loading system into rodents, it was found that stem cells could grow and differentiate normally in hydrogel scaffolds, and the newly formed axons grew along the fiber arrangement of the scaffolds. Subsequently, the team tested the mechanical characteristics of AFG *in vitro* (Cao et al., 2020). The results showed that the AFG could be stretched, knotted, twined, and clipped without breaking (Figure 5C). They further transplanted the AFG loaded with hUMSCs into the semi-resected spinal cord site of the canine. Animal experiments verified that with the help of hydrogel scaffolds, the motor functions of canines were greatly improved in SSCI. From rodents to large animals, the team took a big step in the application of hydrogels in SSCI.

#### 3.3.2 Electrical conductivity

The spinal cord plays an important role in electrical signal transduction in nerve cells. Hence, the reconstruction of electrical conductivity at the SCI site is beneficial for the recovery of spinal cord function (Shu et al., 2019). Zhou et al. (2018) designed a soft, highly conductive, biocompatible CPH based on plant-derived polyphenol, tannic acid (TA), and doping-conducting polypyrrole (PPy) chains. The resulting hydrogels exhibited excellent electronic conductivity (0.05–0.18 S/cm). *In vitro*, high-conductivity CPH inhibits the development of astrocytes and accelerates the differentiation of NSCs into neurons. *In vivo*, CPH has relatively high conductivity, which can activate endogenous NSC neurogenesis in the lesion area, leading to significant recovery of motor function. Similarly, Yang et al. (2022) synthesized an agarose/gelatin/polypyrrole (Aga/Gel/PPy, AGP3) hydrogel for SSCI treatment. PPy, as a conductive material, was doped into the hydrogel to impart high conductivity. *In vivo* studies have shown that AGP3 hydrogel provides a biocompatible microenvironment for promoting endogenous neurogenesis rather than glial fibrosis, leading to significant functional recovery. RNA sequencing analysis further showed that the AGP3 hydrogel regulated the expression of neurogenesis-related genes through the intracellular Ca^2+^ signaling pathway.

#### 3.3.3 Cell affinity

The cell adhesion ability of Arg-Gly-Asp (RGD) peptides is related to the integrins on the cell membrane. Integrins are the main family of cell surface receptors. RGD peptides have been shown to play a central role in adhesion-mediated cell migration required for tissue construction during development and repair. Recent studies have found that around 11 types of integrins can specifically bind to RGD peptides, which are antagonist peptides of integrin receptors (Liu, 2009; Vedaraman et al., 2021). Woerly et al. (2001) designed a poly[N-(2-hydroxypropyl) methacrylamide] (PHPMA) hydrogel containing RGD peptides. RGD peptides were connected to the hydrogels *via* chemical grafting. Experiments have indicated that the PHPMA-RGD hydrogel can reproduce some features of the ECM chemical environment. Similarly, laminin-derived peptides have cell-binding sites that can achieve specific binding. Laminin-derived peptides can chemically react with HA chains to modify the HA hydrogels. For example, Li et al. (2019) reported that laminin-derived peptides PPFLMLLKGSTR could tether HA chains through a reaction between the amino groups of the peptide and the partial aldehyde groups of aldehyde-modified HA. In addition, apart from the RGD peptides and laminin-derived peptides mentioned above, there are some ECM molecules such as collagen, fibronectin, vitronectin, and osteopontin (Onak et al., 2018; Tsiklin et al., 2022) that have corresponding cell binding sites. These peptides can play an important role in cell adhesion when modified into hydrogels.

#### 3.3.4 Injectability

Moreover, it is important to endow hydrogels with injectability. Injectability means that the hydrogel can be injected through a small surgical wound and solidified *in vivo*, which is of great significance for future clinical use. Zhang et al. (2022) developed a facile *in-situ* synthetic strategy for injectable lysine-containing peptide-functionalized hydrogels for the treatment of SSCI. This injectable hydrogel can fill irregular cavities to inhibit glial scar formation and significantly suppress inflammatory responses, thereby further promoting nerve regeneration. Wang et al. (2018) prepared a gelatin-based hydrogel, which was modified using polymer fibers with a shape memory function. The modified hydrogel could recover and maintain the microstructure after injection to a specified position, providing support and guidance for the differentiation of motor neurons. Hong et al. (2017) synthesized amphiphilic injectable poly (organophosphazene) hydrogels and implanted them in animals. *In vivo* experiments demonstrated that the hydrogel system could reconstruct the ECM and improve the microenvironment of SSCI. It contained a hydrophobic group and a hydrophilic group, which gave the hydrogel temperature sensitivity and achieved a temperature-dependent sol-gel transition behavior. The injectability of hydrogels is a promising strategy for *in vivo* implantation in SSCI (Caron et al., 2016; Fuhrmann et al., 2016; Tukmachev et al., 2016; Alizadeh et al., 2020).

#### 3.3.5 Multifunction

Generally, a hydrogel, as a delivery system, possesses more than one function. Yuan et al. (2021) designed a physical dynamic cell-adaptable neurogenic hydrogel (CaNeu hydrogel). The CaNeu hydrogel network stabilized by the reversible ‘host-guest’ complexes was formed by the UV-mediated polymerization of the acryloyl group in acetone washed *β*-Cyclodextrin (Ac-β-CD). Hydrogels have improved physical properties and biological functions, including self-healing properties, mechanical elastic simulation of neural tissue structure and mechanical properties, injectability under gel state, shape remodeling ability, and supporting cell migration ability. The CaNeu hydrogel supported the expansion of adipose-derived stem cells (ADSCs) through the mechanical transmission signal of YAP. The dynamic network of the CaNeu hydrogel provided a permeable ECM for cell migration and growth, thereby promoting axon growth and eventually leading to improved coordination of motor-evoked potential, hind limb strength, and complete spinal cord transection in rats. Luo et al. (2022) prepared natural ECM biopolymer (chondroitin sulfate and gelatin)-based hydrogels for SSCI treatment. This hydrogel delivery system containing polypyrrole, which imparted electroconductive properties, mechanical strength (∼928 Pa), and conductive properties (4.49 mS/cm) are similar to those of natural spinal cord tissues. In addition, the hydrogels exhibited shear-thinning and self-healing abilities, which allowed them to be effectively injected into the injury site and to fill the spinal cord defect segment to accelerate tissue repair in SSCI. Functionalization or chemical modification endows the hydrogels with specific functions. These changes in the characteristics of hydrogels greatly improve their prospects for application in the spinal cord.

### 3.4 Hydrogels synthesized by new methods for SSCI treatment

**FIGURE 6:**
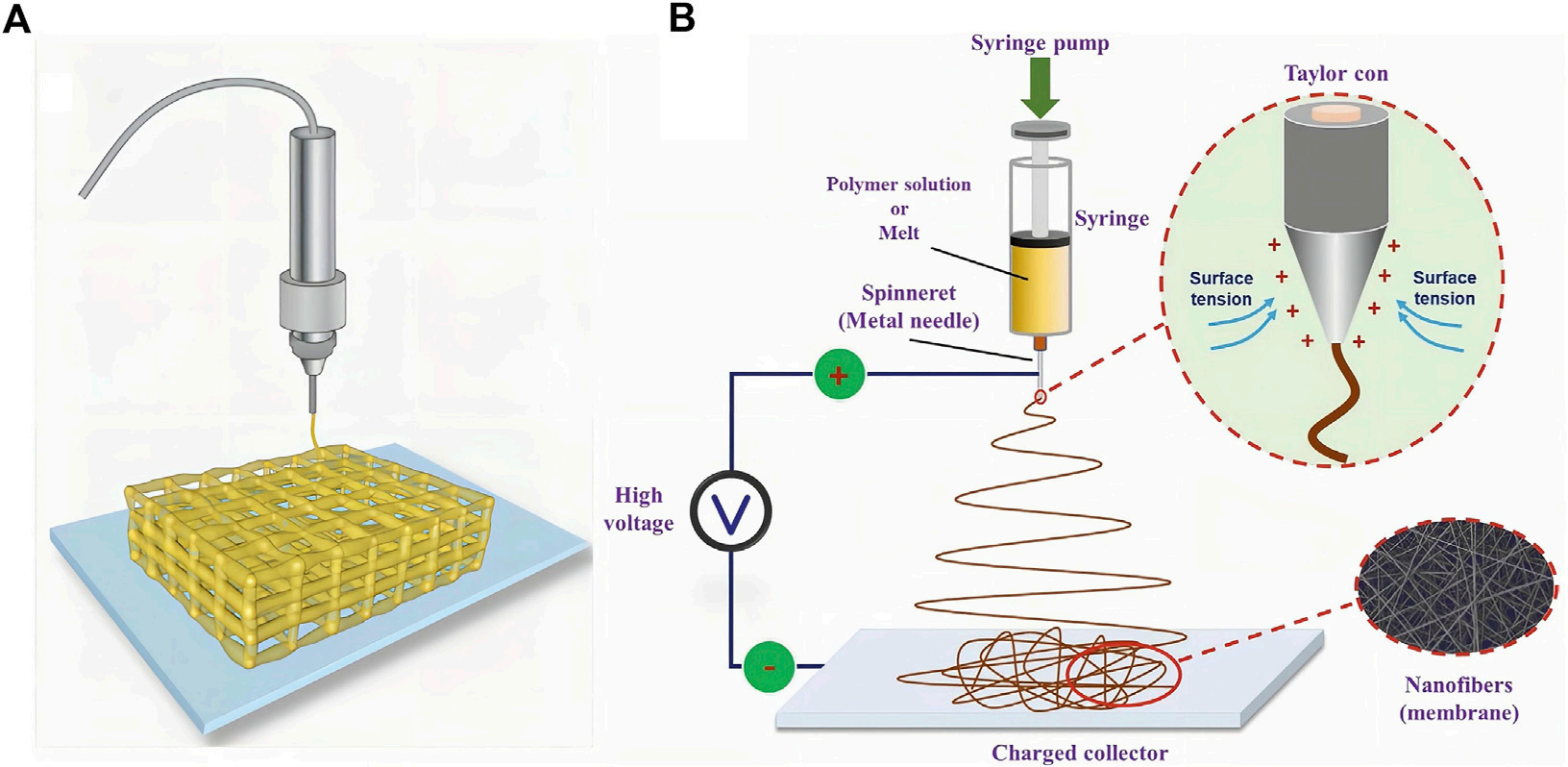
Two kinds of new representative hydrogel fabricating methods. **(A)** Schematic diagram of 3D-printed hydrogel scaffold. Taken from (An et al., 2020). **(B)** Schematic of a conventional electrospinning process with a single jet. [Reprinted with permission from Ref. (Li et al., 2020). Copyright 2020 Wiley].

Hydrogels have a strong drug-carrying capacity and can support cell growth as scaffolds. Owing to the diversity of the internal structure of hydrogels, they have a variety of functions. With the advent of new fabrication methods, hydrogel preparation technology has been constantly updated. We chose two representative new hydrogel preparation techniques for this review: 3D printing and electrospinning. 3D printing is a technology based on digital model files that uses powder metal or plastic and other adhesive materials to construct objects by layer-by-layer printing (Figure 6A) (An et al., 2020). There are some advantages of 3D printing hydrogels. First, the shape, porosity, and other parameters of hydrogels can be accurately controlled by 3D printing technology, improving the embedding degree of hydrogels at the SCI site. Second, NSCs can be added to the bioinks of 3D printing together with nutritional factors or cell growth-promoting drugs, which have great advantages in mass production and quality stability control. 3D printing hydrogel materials usually require excellent fluidity to avoid clogging of printing nozzles and also require good mechanical properties after curing to prevent the collapse of the printed structure (Chimene et al., 2016; Ozbolat and Hospodiuk, 2016). To meet the above requirements, the 3D printing process requires a very short period to achieve ink “sol-gel” *in-situ* transformation (Hölzl et al., 2016). For instance, Li et al. (2021) used a dual-channel 3D printer to print a sodium alginate-Matrigel (SA-MA) hydrogel scaffold, and embedded mesenchymal stem cells (EMSCs) were mixed into the hydrogel solution for printing bioinks. One channel was sprayed with SA-MA, and the other channel was sprayed with CaCl_2_ solution, which required precise control of printing parameters. *In vitro* experiments showed that compared with 2D cell culture, 3D printed three-dimensional scaffolds had better functions to promote the growth and differentiation of EMSCs into neurons. Zhu et al. (2017) selected polyethylene (glycol) diacrylate (PEGDA) as the printing bioink to print a hydrogel scaffold using the stereolithography 3D printing technology. NSCs were loaded onto the scaffold and infrared radiation was used to promote the differentiation of stem cells into neurons. The hydrogel printed by PEGDA was transparent, but its light absorption ability was too strong, so the effect of light was greatly weakened. Therefore, GelMA was added to the bioinks, which greatly reduced the absorption of light; thus, the stem cells loaded on the hydrogel scaffold could receive more red-light irradiation, and the differentiation efficiency was improved. Wei et al. (2016) reported that porous GelMA hydrogels printed by stereo lithography can provide a biocompatible microenvironment for the survival and growth of NSCs. After 2 weeks of *in vitro* culture, the printed structure showed neuronal differentiation and neurite extension. Koffler et al. (2019) further reported that the microscale continuous projection printing method (μCPP) can be used to produce hydrogel scaffolds that fit the spinal cord structure. This printing method can improve the integrity of hydrogel scaffolds and enable faster printing.

Electrospinning, a widely used technology for electrostatic fiber formation, utilizes electrical forces to produce polymer fibers with diameters ranging from 2 nm to several micrometers using solutions of both natural and synthetic polymers (Figure 6B) (Ahn et al., 2006; Alessandrino et al., 2008; Li et al., 2020). Electrospinning technology involves simple manufacturing equipment, low spinning cost, a wide variety of spinnable materials, controllable processes, and diverse spun structures. They can also be used to fabricate hydrogels. The main requirements of the electrospinning technology for raw materials are that the polymer solution must have a suitable concentration and viscosity. In addition, the uniformity and stability of the solution should be guaranteed when preparing electrospinning solutions (Ahn et al., 2012). For example, Cao et al. designed a hierarchically aligned fibrin hydrogel through electrospinning, and the cells grew along with the aligned fibrin (Yao et al., 2018). In addition, hydrogels with different structures can be synthesized by electrospinning. He et al. (2018) fabricated an antimicrobial peptide-loaded gelatin/chitosan nanofibrous membrane using layer-by-layer electrospinning and electrospraying techniques. Electrospraying can be interspersed with electrospinning technology to uniformly spray drug-loaded nanospheres or other forms of particles into multilayer structures, which is beneficial for carrier design and drug release. Zang et al. (2017) prepared a pH-responsive nanofibrous hydrogel membrane using uniaxial electrospinning. Electrospinning can be combined with other raw materials to fabricate a variety of fiber-based structures. Zhang et al. (2021) synthesized fiber-hydrogel scaffolds through electrospinning. First, the electrospinning solution was pre-treated. Aligned PCL fibers were then synthesized using a two-pole airgap electrospinning technique. Finally, collagen was used to form the hydrogel matrix surrounding the fiber bundles according to the protocol.

Both 3D printing and electrospinning can accurately form hydrogel skeletons and synthesize complex hydrogels. The precise structures of hydrogels are conducive to the loading of active materials. For example, cells can grow along the direction of the scaffold, which significantly improves the therapeutic effect after implantation. With the continuous development of these emerging technologies, the clinical application of hydrogels in SSCI treatment will be further improved.

## 4 Perspective and conclusion

SSCI is an important cause of irreversible injury, which includes the excessive release of glutamate, causing cell and tissue toxicity, followed by ROS and lipid peroxidation, further immune and inflammatory reactions, and subsequent glial scar formation. However, these reactions are detrimental to nerve cell regeneration. Thus, the treatment of SSCI should focus on the differentiation of NSCs and the improvement of the local cell growth microenvironment. Currently, the local delivery of nerve protection and neurotrophic factors, stem cell differentiation factors, drugs or other bioactive materials is an important way to improve the microenvironment of SSCI. Meanwhile, the hydrogel scaffold could guide the axon growth direction of nerve cells, which promoted the connection between new axons and host residual nerve cells. Owing to their appropriate mechanical strength, the structure of the oriented arrangement, and excellent load-bearing capacity, hydrogels can act as excellent delivery systems for SSCI treatment.

In this review, hydrogels are divided into two parts: natural hydrogels and synthetic hydrogels. Natural hydrogels have inherent excellent biocompatibility and mechanical properties; however, they still have some limitations. A variety of natural hydrogels differ in their structures, degradation properties, and mechanical strengths, and fine and rigorous operation is required in the synthesis process. Unlike natural hydrogels, synthetic hydrogels can select the corresponding raw materials according to the demand, and most of the raw materials used for the synthesis of hydrogels are renewable with low manufacturing costs. However, their long-term biocompatibility may not be as good as that of natural hydrogels. In addition, it is common to use more than one raw material for hydrogel preparation. For example, Geissler et al. (2018) synthesized biomimetic hydrogels composed of collagen, hyaluronic acid, and laminin from three natural biological materials. After implantation in rats, the differentiation of oligodendrocytes into NPCs was significantly increased. Meanwhile, rats with SSCI after hydrogel transplantation showed functional recovery with or without NPCs. Compared to the control group, the functional recovery of animals transplanted with the NPCs hydrogel was significantly increased within 6 weeks. The results showed that hydrogels synthesized using various raw materials combine the advantages of each material, although the synthesis process may be more complex. In general, natural hydrogels are more likely to be used for SCI delivery than synthetic hydrogels based on their biocompatibility and biofunctions. However, some synthetic polymers can be added to natural hydrogels to enhance their mechanical properties or to change their degradation time; thus, the preparation of hydrogels by combining natural and synthetic materials is a promising method. Alternatively, some advanced methods are developed in hydrogel fabrication. We introduced two new technologies: 3D printing and electrospinning. These methods varied the hydrogel fabrication process and broadened the application of hydrogels in biomedical applications.

When drugs, cytokines, and stem cells are delivered through hydrogels to the SCI site, their release is closely related to the rate of hydrogel degradation. Moreover, the release rate of traditional and cytokine drugs in hydrogels can be controlled. If the drug can be first coated with nanoparticles, the nanoparticle shell can still release the drug even after the hydrogel is degraded. As previously mentioned, the introduction of chitosan nanoparticles can extend the release time of the drug (Mahya et al., 2021). In addition, in the design of hydrogels, the presence of a porous structure affects the release rate of the drug in the hydrogel, and the pore size of the hydrogel network structure also affects the release rate of the drug (Siboro et al., 2021). Moreover, drugs can form a highly stable covalent connection with the raw materials of the hydrogel, thereby prolonging the release of the drug until network degradation. These factors should be considered when designing a hydrogel delivery system to achieve on-demand drug release (Li and Mooney, 2016).

In SSCI treatment, hydrogels with some functions, such as stronger adhesion, shape memory, antioxidant capacity, and injectability, are beneficial to therapeutic effects. However, there are still some combined treatment methods in SSCI therapy research. For instance, hydrogel-loaded stem cells have been combined with infrared light to stimulate nerve cell regeneration (Li et al., 2021). Liu et al. (2021) promoted nerve cell regeneration through the delivery of nerve growth factors using a conductive hydrogel combined with electrical stimulation. *In vitro* and *in vivo* experiments have verified that a combination of physical stimulation can achieve better functional recovery of the spinal cord. These combination therapies warrant further investigation. This broadens the application of hydrogels in SSCI and shows good prospects for future clinical applications.
